# Nitrate of Silver in Toothache

**Published:** 1858-03

**Authors:** J. S. Dixon

**Affiliations:** Ashland, Cheatham County, Tenn.


### ARTICLE IV.

*Nitrate of Silver in Toothache.* By J. S. DIXON, M.D., Ashland, Cheatham County, Tenn.

In the January number of your Journal, I see mention made of the use of nit. arg. in carious teeth. I have been using it in my practice for several years, and have used it in my own teeth when they were aching with decided relief. It can be used with less inconvenience than fluids. I generally place a piece about the size of a grain of mustard seed on a lump of wax, and then place it in the cavity of the tooth, and seal it over with the wax. It will destroy the nerve of the tooth.

